# Technology Priorities for Diabetes Care in Rural Georgia: Findings of a Problem Discovery Workshop

**DOI:** 10.5888/pcd23.260489

**Published:** 2026-11-12

**Authors:** Ruwanthi N. Ekanayake, Lynn Glenn, Shelley Spires, Henry N. Young, Michael R. Kramer, Rudy Gleason, Jithin Sam Varghese

**Affiliations:** 1 Global Health and Development Doctoral Program, Laney Graduate School, Emory University, Atlanta, Georgia; 2 Medical Scientist Training Program, School of Medicine, Emory University, Atlanta, Georgia; 3 Augusta University College of Nursing , Department of Nursing Science, Augusta, Georgia; 4 Albany Area Primary Health Care, Albany, Georgia; 5 Department of Clinical and Administrative Pharmacy, College of Pharmacy, University of Georgia, Athens, Georgia; 6 Center for Rural Health and Health Disparities, Mercer School of Medicine, Mercer University, Macon, Georgia; 7 George W. Woodruff School of Mechanical Engineering, Georgia Institute of Technology, Atlanta, Georgia; 8 Wallace H. Coulter Department of Biomedical Engineering, Georgia Institute of Technology and Emory University, Atlanta, Georgia; 9 Institute for People and Technology, Georgia Institute of Technology, Atlanta, Georgia; 10 Hubert Department of Global Health, Rollins School of Public Health, Emory University, Atlanta, Georgia; 11 Emory Global Diabetes Research Center, Woodruff Health Sciences Center, Emory University, Atlanta, Georgia

## Introduction

In the US, the rural South faces a disproportionate population health impact from diabetes: while national diabetes mortality has generally declined since 1999, the prevalence of diabetes in rural areas is 17% higher than in urban areas, and mortality rates in the rural South dropped by less than 1 death per 100,000 between 1999 and 2016 (1). Faced with rapidly closing local hospitals and physician shortages (2–4), the rural South must find new ways to address this crisis.

Rapidly advancing health technologies such as remote patient monitoring systems and artificial intelligence–assisted interventions present exciting possibilities. However, many such interventions fail to account for rural social and cultural contexts. People in rural areas have varying levels of digital literacy, face stigma around help-seeking, and trust intimate networks over institutions for medical guidance (5). These contexts, along with other unknown and unmeasured factors (eg, competing caregiving/work demands), can impede patient engagement with health technologies, leading to suboptimal intervention outcomes (6,7).

A problem discovery approach identifies and characterizes health needs before solution design (8). This approach, often featuring discussion-based workshops that engage community members and subject matter experts, has been useful in creating tailored interventions in low-resource communities with limited health care access (9). In the rural South, a problem discovery approach can leverage community engagement to identify heterogeneous and tacit local needs that may be underrepresented in published literature and thereby result in more relevant and viable interventions.

We convened rural health subject matter experts (SMEs) from various domains (eg, clinical medicine, administration, information systems) for a problem discovery workshop in Macon, Georgia, modeled on previous workshops organized by the James Lind Alliance and Global Network of WHO Collaborating Centres for Bioethics (10,11). Through structured exercises on ideation and consensus-building, SMEs identified and characterized 10 technology priorities (ie, digital tools, devices, and technology-enabled approaches to prevention, diagnosis, monitoring, or treatment) for diabetes care in rural Georgia.

### Recruitment of workshop participants

We (the academic working team) partnered with a steering committee of rural Georgia leaders in clinical practice, academic research, health care delivery, law, and regulatory compliance. The steering committee guided our selection of SMEs from rural health networks, advised on cultural and professional guidelines for engaging with SMEs (including norms for respectful communication and relationship building), and helped facilitate the problem discovery workshop.

In selecting SMEs, we prioritized diversity of experience, long-standing service to rural Georgia populations, and interest in novel health initiatives. Fourteen SMEs attended the workshop, representing clinical medicine, nursing, pharmacy, mental health, nutrition, public health, health care operations, laboratory science, and health technology. 

In addition to SMEs, we recruited workshop observers with backgrounds in technology, education, strategy, and public health from the networks of the academic working team and the steering committee. Although observers did not have rural health experience, they were asked to promote multidisciplinary conversation by participating in discussions without casting deciding votes, asking clarifying questions, and introducing ideas for SME reflection (eg, mentioning a particular technology to see if it might meet a need raised by an SME). 

### Workshop design, planning, and execution

Workshop design and planning occurred from January through June 2025. Because the workshop did not involve human subjects, institutional review board approval was not required.

The workshop was grounded in principles of human-centered design, problem discovery theory, and the Delphi methodology, with the goal of moving from unrestricted ideation to consensus (12,13).

The workshop took place in Macon, Georgia, at the Mercer University Innovation Center. It was supported by scribes and note synthesizers. SMEs participated in journey-mapping and “brain-writing” exercises, transitioned into small and large group discussions to refine a penultimate list of technology priorities for diabetes care in rural Georgia, and then ranked these priorities in an anonymous mobile phone-based Qualtrics survey (Qualtrics, LLC). Finally, SMEs ratified the top 10 priorities in a final discussion and completed an anonymous feedback survey.

In the months after the problem discovery workshop, the steering committee and academic working team used the final 10 technology priorities and workshop-generated context to develop a set of public briefs to inform innovation.

## Workshop Findings

### Common themes and design principles

Several common themes, design principles, and development specifications emerged from the final 10 technology priorities for diabetes care in rural Georgia.

**Building for limited connectivity.** SMEs emphasized that many rural Georgia patients do not have reliable access to cellular service and that rural broadband expansion is a long and financially prohibitive process. Useful workarounds include offline capability and storage for essential functions, central system syncing, leveraging local health centers with data coverage, and backward compatibility with widely-available technology options (eg, SMS, telephone calls).

**Digital literacy and user experience.** SMEs stated that many rural Georgia patients often prefer to interact with health care providers rather than with digital tools. Technology-driven solutions should prioritize user experience as well as strategies that will encourage rural Georgia patients to use the technology and should identify which processes are appropriate for digitization or for personal contact.

**Relationship-focused co-design.** SMEs highlighted that many rural Georgians have a strong sense of community. SMEs stated that technologies that leverage the support of trusted community touchpoints (eg, churches, community centers, hair and nail salons) were more likely to succeed.

### The top 10 technology priorities for diabetes care

The top 10 technology priorities in rural Georgia spanned 4 domains: 1) clinical practice and diagnosis, 2) care delivery systems, 3) medication access and adherence, and 4) nutrition and lifestyle support (Table).

**Table:** Top 10 Technology Priorities for Diabetes Care in Rural Georgia, 2025^a^

Domain	Technology priority
Clinical practice and diagnosis	1. Develop integrated decision-support tools in electronic health records to help rural primary care providers accurately diagnose and manage diabetes.
	2. Develop a streamlined system for rural clinics that tracks laboratory specimens in real time, ensures adherence to diverse laboratory protocols, and prevents errors such as misrouting, sample expiration, and data loss.
Care delivery systems	3. Enable rural primary care providers to manage complex diabetes cases by providing specialist support and technology-enabled care coordination between providers.
	4. Enable care coordination that connects clinicians, pharmacists, diabetes educators, and community health workers to deliver holistic diabetes care in rural settings.
Medication access and adherence	5. Develop a simplified, unified system for prescribing and securing prior authorizations for advanced diabetes therapies (eg, GLP-1s, insulin, continuous glucose monitoring devices).
	6. Develop and implement culturally concordant, easy-to-understand learning tools to reduce medication discontinuation from side effects among patients with diabetes in rural areas. Enable rural health care providers to track medication adherence, receive real-time alerts, and identify patients at high risk of disease progression due to severity of disease (eg, glycated hemoglobin A_1c_ >9%), facilitating timely outreach and care coordination without increasing provider workload.
Nutrition and lifestyle support	7. Equip local clinics with tools and training to deliver culturally relevant, accessible diabetes nutrition education during routine visits.
	8. Empower patients with culturally relevant, practical tools and community-based support to sustain healthy eating habits beyond the clinic, rooted in their daily routines, food environments, and social networks.
	9. Support the development of community-driven programs that use local spaces and/or virtual coaching to overcome safety and transportation barriers, increasing safe physical activity for patients with diabetes in rural areas.
	10. Provide personalized, ongoing stepwise learning for patients adjusting to a new diabetes diagnosis that addresses and monitors mental health effects and aligns with each patient’s readiness.

a The source for the information is a group of invited rural health subject matter experts from Georgia. The group produced the list of technology priorities at a problem discovery workshop held in Macon, Georgia, in 2025. A detailed report is available at www.stopncd.org/top-10-problems/rural-georgia.

#### 1. Clinical practice and diagnosis

SMEs noted that rural Georgia primary care providers, many of whom are recent medical school graduates with low levels of peer support, are often overwhelmed by patient volume, lack experience initiating new diabetes regimens, and are undertrained on distinguishing type 2 diabetes from atypical presentations of type 1 diabetes. Suggested areas for technological support included guidance on risk-stratified treatment pathways and type 1 diagnostics (C-peptide, islet autoantibody testing) and on-demand educational modules on patient counseling.

Many rural clinics have limited laboratory staff and rely on external laboratories for processing specimens. Laboratory compliance errors (mislabeling, improper storage) lead to expensive repeat testing and delayed diagnoses. SMEs therefore also highlighted the need for streamlined support for adherence to laboratory protocol, real-time tracking of specimens, and timely correction of sample errors.

#### 2. Care delivery systems

Many patients in rural Georgia face substantial hurdles to care: months-long waitlists, transportation barriers, and inconsistent transfer of medical records. Most health care providers are not able to engage in cross-specialty communication (eg, primary care physicians working with endocrinologists, ophthalmologists, neurologists, and nephrologists to provide comprehensive care), leaving patients to navigate complex care protocols by themselves.

SMEs identified multiple areas for innovation, including enabling rural primary care providers to manage complex diabetes cases with specialist support and technology-enabled care coordination between providers.

#### 3. Medication access and adherence

Rural Georgia patients face many challenges in accessing medication. One is prior authorization attempts that come with a costly fee whether approval for the medication is granted by the health insurance company or not. Poor interoperability with state Medicaid formularies and pharmacy software prevents timely verification of coverage and cost.

Additionally, patients often receive rushed, limited education on diabetes medications in understaffed rural clinics. Without being prepared for unpleasant side effects of diabetes therapeutics (nausea, vomiting, bloating), many discontinue the medications.

SMEs emphasized the need for enhanced systems for prior authorizations and medication cost verification, auto-enrollment in financial assistance programs like the federal 340B Drug Pricing Program (14), culturally concordant patient medication education, and medication adherence tracking for patients with severe disease at highest risk of progression (eg, glycated hemoglobin A_1c_ >9%).

#### 4. Nutrition and lifestyle support

Although lifestyle modifications have proven benefits in managing diabetes, rural health systems have not maximized strategies to achieve lifestyle modifications. Patients receive generic in-clinic nutrition counseling, feel reluctant to compromise community food traditions, and struggle with feeling isolated, overwhelmed, and anxious. Environmental constraints include geographic and financial inaccessibility of exercise options (few gyms and green spaces) and fragmented community resources (food pantries, County Extension health initiatives).

SMEs emphasized the need for technology solutions for culturally relevant mental health support, actionable lifestyle modifications, and connections with community support resources.

### Feedback on the workshop from subject matter experts 

SMEs commented favorably on the quality and diversity of discussions and reported excitement about potential future collaborations. Suggested improvements included expanded reflection time and participant pool and a more user-friendly survey form for ranking priorities.

## Discussion

Two overarching themes throughout the workshop were that 1) digital access and literacy are limited for many patients and 2) in-person relationships and community have high value in rural Georgia. SMEs emphasized that even if connectivity issues are fully resolved, personal interactions must be preserved when any technological solution is used.

Strengths of the workshop include community-based design, experts representing interdisciplinary domains, and a strong focus on problem discovery and expansive ideation. Limitations included its short duration (1 workday), SME selection being limited to the steering committee’s network, lack of patient participation, and user fatigue with the mobile telephone–based Qualtrics survey. Future efforts can include a broader range of SMEs and patients and a more user-friendly problem ranking platform.

The importance of community and network-building in technology innovation was highlighted by the problem discovery workshop. Organization and execution of the workshop and the insights offered by the SMEs were possible only because of extensive collaboration with community partners. Thus, the most valuable output of a rural health technology initiative may not be the software, but rather, the partnership.
